# Supplementary material for: The Effects of Silencing PTX3 on the Proteome of Human Endothelial Cells
Source: Int J Mol Sci. 2022 Nov 3;23(21):13487. doi: 10.3390/ijms232113487 (PMC9654901; doi:10.3390/ijms232113487)
Supplement: Supplementary file 1 [file ijms-23-13487-s001.zip › Figure S1.pdf]

Lanes 3/4 have been cropped for Fig. 3a (KDR).
Lanes 1 to 8 have been used for quantification (Fig. 3b).

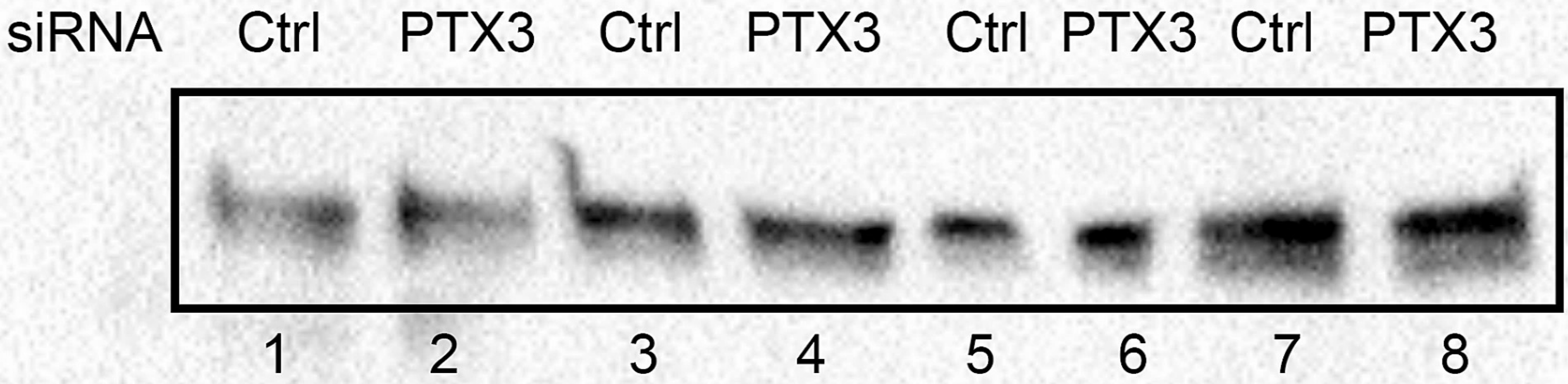

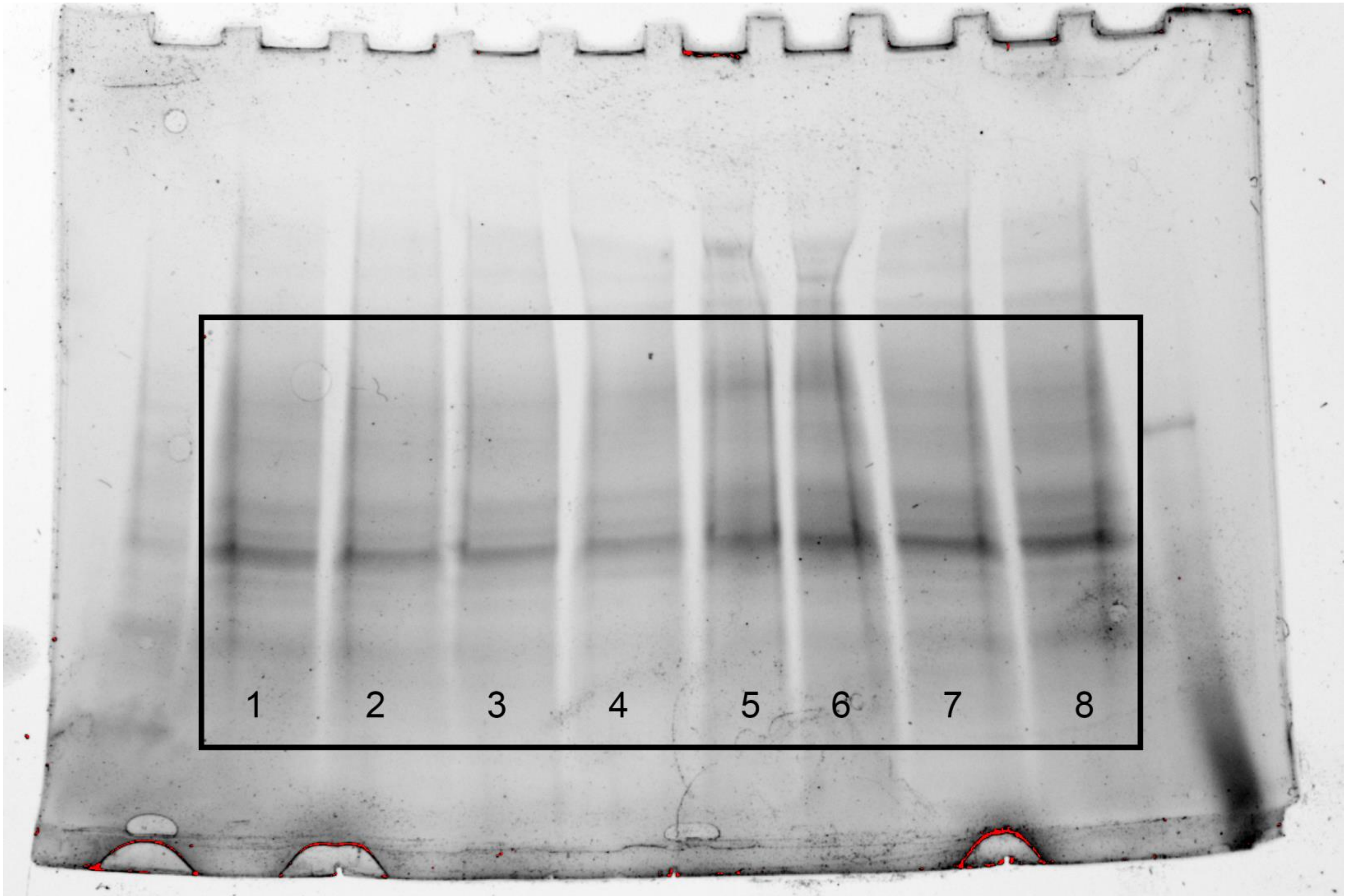

Lanes 3/4 have been cropped for Fig. 3a (KDR, tot prot).
Lanes 1 to 8 have been used for quantification (Fig. 3b).

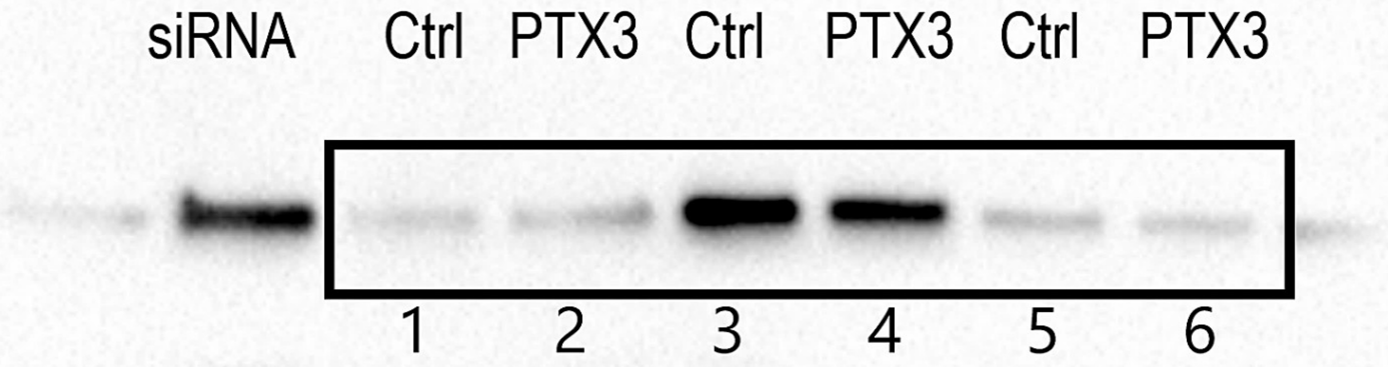

Lanes 3/4 have been cropped for Fig. 3a (eNOS).
Lanes 1 to 6 have been used for quantification (Fig. 3b).

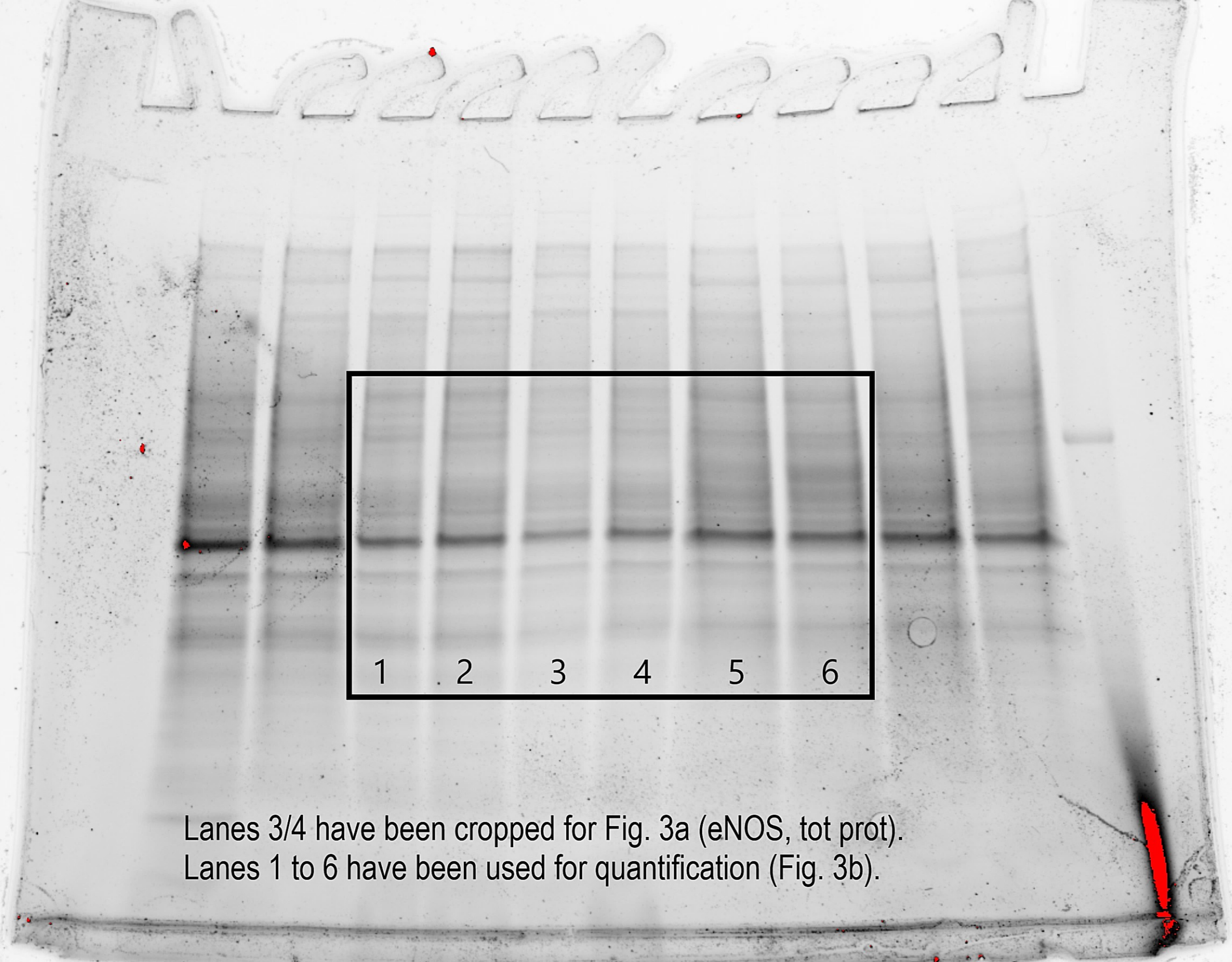

Lanes 3/4 have been cropped for Fig. 3a (eNOS, tot prot).
Lanes 1 to 6 have been used for quantification (Fig. 3b).
