# Supplementary material for: The Effects of Silencing PTX3 on the Proteome of Human Endothelial Cells
Source: Int J Mol Sci. 2022 Nov 3;23(21):13487. doi: 10.3390/ijms232113487 (PMC9654901; doi:10.3390/ijms232113487)
Supplement: Supplementary file 1 [file ijms-23-13487-s001.zip › Table S1.pdf]

Table S1. Functional enrichments of Biological Processes in the network created by STRING with the proteins more abundant in PTX3 silenced cells.

#Term ID	Term description	Observed gene count	Background gene count	Strength	False discovery rate	Matching proteins in your network (labels)
GO:0000398	mRNA splicing, via spliceosome	6	294	1.24	0.0128	SRSF9, SNRPC, U2AF2, PSPC1, SNRPB, ALYREF
GO:0045765	Regulation of angiogenesis	6	303	1.23	0.01280	GRN, ITGA5, ATP2B4, EPHA2, ENG, ANXA1
GO:0001649	Osteoblast differentiation	4	127	1.43	0.0254	CCDC47, EPHA2, RRBP1, ALYREF
