# Supplementary material for: The Effects of Silencing PTX3 on the Proteome of Human Endothelial Cells
Source: Int J Mol Sci. 2022 Nov 3;23(21):13487. doi: 10.3390/ijms232113487 (PMC9654901; doi:10.3390/ijms232113487)
Supplement: Supplementary file 1 [file ijms-23-13487-s001.zip › Table S2.pdf]

Table S2. List of primers for human genes. For custom primers, forward and reverse sequences are reported. For commercially available primers, the Qiagen codes are indicated.

**Gene**	**Sequences**
GAPDH	Forward: ACG GAT TTG GTC GTA TTG GGC<br>Reverse: CTC CTG GAA GAT GGT GAT GG
PTX3	Forward: TGC GAT TCT GTT TTG TGC TC<br>Reverse: TGA AGA GCT TGT CCC ATT CC
EPHA2	QT00028595
ENG	QT00013335
PTGS1/COX-1	QT00210280
IL1-β	QT00021385
IL-6	QT00083720
MCP-1	QT00212730
